# How Should Dentists Advise People to Clean the Teeth?

**Published:** 1915-12

**Authors:** Bernard Feldman

**Affiliations:** Perth Amboy, N. J.


### HOW SHOULD DENTISTS ADVISE PEOPLE TO CLEAN THE TEETH?

BY BERNARD FELDMAN, D.D.S., PERTH AMBOY, N. J.

---

At the dedication exercises of the Evans Institute held this year in Philadelphia, clinics were given by several prominent men on various topics. One clinician expounded his views to the dentists surrounding him as to what he considered to be the right way for people to clean the teeth. He advocated the vigorous use of the tooth brush on the gums and teeth. At another chair, another clinician was very emphatically advising his hearers that the bristles of the brush should *not* touch the gum tissue, as such brushing action was decidedly injurious to the free margins and soft tissues. This was the sole reason for his clinic—to warn the dentist against advising his patient to use the bristles of the tooth brush on the gum tissue while cleaning the teeth. Standing nearby and listening intently was the writer, possessing just as sincere and honorable intentions to advise the best method of cleaning the teeth, and having even more radical views on this matter. These views were shortly afterward published in the March 1915 issue of *Oral Hygiene*—The Menace of the Tooth Brush, and reprinted partly in the *Literary Digest*, May 22.

The average person takes little if any care of his tooth brush; and it can be safely said that the brushes, as used by over ninety per cent. of the public, are filthy germ-carriers, which are doing more harm than good in preventing caries and pyorrhea. A close study of the matter will convince any impartial person (who is eager to see the truth, however unpleasantly it may affect a time-worn tradition as the brush has already become) that the brush does not reach the interproximal spaces to dislodge the food-remains efficiently; that it cannot be sterilized efficiently without ruining the bristles or handles; that it tears and scrubs the soft tissues of the mouth and that it is undesirable in principle to use the same kind of agent that is being used to clean floors and gutters, to clean the oral cavity. Of course the tooth brush has smaller and finer bristles, and a handle to conform to the size and shape of the mouth; but the principle is identical in all brushes used for cleaning purposes.

It will be argued that there are any number of people who have taken care of their teeth using the tooth brush for scores of years, and that these teeth are good and healthy. There was a time when people would clean their rugs and carpets with the old-fashioned broom, never realizing the wear-and-tear that was being caused by the bristles. Nevertheless, no one will deny that the vacuum cleaner of today will clean the dirt off the rug much more efficiently, with less wear-and-tear and less discomfort. No one will take the stand that this decided improvement of the electric vacuum cleaner is to be frowned upon; yet there are many people who are still sweeping their rugs with the broom.

Any technique that had for its purpose the cleanliness of the oral cavity was a step in the right direction. But if an improved method can be devised and truthfully established, which will conform more closely to Nature's wishes of sanitation and health, then it is the duty of the dental profession to cast aside all traditions as to the gospel of the tooth brush, to forget that this universal custom has become so popular as to be considered almost gospel truth, and to turn instead, to this improved method of cleaning the oral cavity. There should be no let-up in drilling the children and educating the masses to the *right* methods of mouth hygiene; these efforts are certainly to be praised and assisted. But when children and masses are being taught a harmful habit, in the belief that it is oral prophylaxis, the writer says "Stop! Has this mistake not done enough harm? Why continue it, when an efficient method could be advised by an impartial commission of research workers who would investigate the subject?

"Let there be but one method of cleaning teeth which could be advocated uniformly by every dentist; but let that one way be a right way. If the tooth brush is really efficient, let the dentists of the world become teachers of tooth brush drills; if the brush is proved to be harmful let this wild enthusiasm stop, and an attempt made to stem the tide and turn it the other way."

It has been squarely put up to the dental profession, "Will it take the next step in civilization," by effectively taking care of the mouth. The nose and pharynx are comparatively sterile; the nose, in fact, is a protection against infection. It can be safely inferred that the mouth is the greater culture ground for all sorts and varieties of germs, it naturally follows that the cleaning of this focus becomes the greater factor in the problem of disease prevention. Preventive medicine will find in mouth cleanliness the greatest weapon with which to stem the ravages of disease for future humanity. It is, therefore, a source of regret that dentists should be divided over this important question, resulting in conflicting ideas being presented to the public, in the dentists' sincere and earnest desire to render the best dental service. It is also regretted that a research commission has not been appointed to settle the question, "What is real mouth hygiene?" Thus far, the public has been "educated" by full-page advertisements to believe that chewing gum, lozenges, oxygen powder, tooth brushes, etc., are "good for the teeth." The great influence which this heavy advertising campaign has wielded upon the public mind can only be guessed at.

Being intensely interested in this subject, the writer has carefully investigated the entire field, and has been astonished that so many apparently obvious things are being overlooked in mouth hygiene. The question of dentifrices and mouth washes has not been given the serious consideration it deserves; what little research work has been done in this matter being given little if any heed. The importance of diet as a prophylactic agent has never been seriously considered. The matter of fruit-juices, vinegar washes and acid powders surely deserve closer study than has been given them. The tooth brush use is not the only custom that is so universally popular and so undeniably harmful. For instance, it has occurred to many people that antiseptic mouth preparations are too strong and irritating to the soft tissues of the mouth. Doctor Ebersole, the father of the mouth hygiene movement of this country, said in a personal communication, "I am unqualifiedly opposed to the use of antiseptics and disinfectants in the mouth, because of the fact that these agents attack the superficial cells, the soft tissues of the mouth, more readily than they attack and destroy the disease producing germs. Therefore, a mouth wash or dentifrice and the method employed in using the same can only be effective in proportion to its ability to loosen up and wash out the disease producing germs and debris found in the mouth."

Doctor McGhee, of Cincinnati, experimented with the more popular dentifrices that are on the American market. He found that almost without exception, the coloring matter (which was added for no medicinal value, but to promote a ready sale), had a decided tendency to discolor the teeth. Cracks in the enamel and cavities of decay furnished the avenues of entrance for stains of all kinds to penetrate the enamel, in spite of the dense structure and the chemical affinity which enamel has for other substances. These research findings are important; nevertheless, little mention has been made by writers and lecturers, condemning the practice of people using colored dentifrices to clean their teeth with. These dentifrices depend for their efficiency upon a strong antiseptic drug to attack the germs and food debris to thus obtain mouth cleanliness. It is ridiculous to expect that this chemical reaction will prove beneficial to the tissues. Firstly, it is very irritating to the cells of the mucous membrane of the mouth, and instead of assisting them to a healthy growth, there is a degeneration of the cells which leads to their death. Secondly, the only way to remove food debris is to do it mechanically by means of the silk floss or a metal strip or a piece of cotton gauze.

The writer maintains that the nearer Nature's wishes are approached, the sooner will her processes of health and repair be solved. The mouth is peculiarly adapted to receive and to hold germs and food debris. The thirty-two teeth, their tooth sockets, the interproximal spaces, the tonsils, the faulty fillings and dentures, the tongue, etc., offer avenues of infection from the mouth to the general system. To clean this big focus in the way that will conform closest to Nature's wishes, the problem must therefore be studied systematically with the idea of cleaning these various avenues of infections. The writer wishes to submit his six principles of mouth hygiene, which constitute a rational routine to obtain mouth cleanliness.

(1) Taking care of what one eats, and how one eats it.

(2) Removing mechanically all food deposits.

(3) Keeping the surfaces of the teeth polished; dental service must be sanitary.

(4) Gently massaging the gums and outer surfaces of the teeth.

(5) Carefully washing the mouth with a mild saline solution or plain water.

(6) Regular prophylactic service by the dentist.

The greatest factor is the mechanical removal of food debris between the interproximal spaces without hurting the gum tissues. This object can be best obtained by the dental silk floss or metal strip or a strong liquid spray. To subject the mouth daily to strong drugs, when the very cause of tooth-decay, *i. e.*, food debris, is allowed to remain, is retarding Nature's processes instead of healing them. Healthy tissue requires gentle massage and careful cleansing; the massaging adds tone to the gums by creating antibodies to diseases, and the cleansing with a mild restorative will thereupon keep the mouth clean and wholesome. It is true that bacteria play an important part in the process of tooth-decay; but their action is secondary. "Remove all deposits, and there is no pabulum for the bacteria to thrive upon; keep the interproximal spaces and outer surfaces of teeth polished, and they can not obtain their initial hold on the tooth."

The dental floss must be intelligently used, as it may harm the gums. Doctor Ebersole says, "my faith in the use of the floss, has been so thoroughly shaken that I hesitate to give a demonstration of how the floss should be used. Some of us have been investigating the matter thoroughly and have been convinced of the inadvisability of placing the floss in the hands of the general public." Although the writer hesitates to take such a radical stand, he prefers the metal strip as the agent par excellence to polish the interproximal spaces. Care must, of course, be taken not to injure the gum tissue. Dr. G. V. Black claims that a mouth can be effectually cleansed with a spray of normal saline solution. Any colorless and weak solution that will promote a healthy flow of saliva will be found to have a healthy effect upon the tissues. Plain water seems to be Nature's own mouth wash to clean the mouth. It is self-evident that regular prophylactic service must be given to make certain that slight abnormal conditions are prevented from causing further serious harm.

Concluding, in plain words, the following is the method by which the writer advises the public to keep the teeth clean:—Exercise the teeth by eating the right kind of food in the proper way. The savage, it has been claimed, owed his splendid denture to his diet. After each meal, but most particularly before going to bed, one should try to remove the food remains in a rough way by washing the mouth with plain water, playing the lips and the cheeks upon the tooth-surfaces, finally gargling the water. The remainder of the food is then mechanically removed by passing silk floss or preferably a thin strip of thin metal between the teeth, to clean the interproximal spaces. The clean forefinger is then covered slightly by a piece of cotton gauze or rubber, and the outer surfaces are "dry rubbed" to remove all stains of deposits, that may be attached near the gum margins. The clean fore-finger can then be used to massage the tissues with a colorless and weak dentifrice, one that will not discolor the teeth or prove too irritating to the tissues. The clean forefinger thus becomes Nature's own tooth brush, which "just fills the bill." The excess antiseptic is then washed out with plain water, or a weak saline solution. A spraying device is also beneficial. The dentifrice advocated by the National Mouth Hygiene Association, called Mogene, has been carefully compounded and should be recommended by the dentists for the double purpose of giving the patient the very best dentifrice, and secondly, for supporting the educational features of the Association from the revenue derived through the sale. Regular prophylactic service, which will include good sane advice as to healthy habits, is essential. Anything that will tend to make the blood richer or that will add tone and vitality to the body will have a tendency to make even the teeth sparkle with the glow of good health. The person with the "mouth beautiful" gradually acquires a vital resistance that is a great factor in the maintenance of his good health.—*Dental Digest.*

